# Converting Corncob to Activated Porous Carbon for Supercapacitor Application

**DOI:** 10.3390/nano8040181

**Published:** 2018-03-21

**Authors:** Shaoran Yang, Kaili Zhang

**Affiliations:** 1 Department of Mechanical and Biomedical Engineering, City University of Hong Kong, Kowloon, Hong Kong, China; shawnyang_hk@163.com; 2 Shenzhen Research Institute, City University of Hong Kong, Shenzhen 518057, China

**Keywords:** biowaste, hierarchical porous carbon, KOH activation, symmetric supercapacitor

## Abstract

Carbon materials derived from biomass are promising electrode materials for supercapacitor application due to their specific porosity, low cost and electrochemical stability. Herein, a hierarchical porous carbon derived from corncob was developed for use as electrodes. Benefitting from its hierarchical porosity, inherited from the natural structure of corncob, high BET surface area (1471.4 m^2^·g^−1^) and excellent electrical conductivity, the novel carbon material exhibited a specific capacitance of 293 F·g^−1^ at 1 A·g^−1^ in 6 M KOH electrolyte and maintained at 195 F·g^−1^ at 5 A·g^−1^. In addition, a two-electrode device was assembled and delivered an energy density of 20.15 Wh·kg^−1^ at a power density of 500 W·kg^−1^ and an outstanding stability of 99.9% capacitance retention after 4000 cycles.

## 1. Introduction

The recent growing demand for portable electronic devices and electrical vehicles have urged researchers to develop effective and sustainable energy storage systems. Supercapacitors, as one of the most promising energy storage devices, have attracted considerable attention for their fast recharge ability, high power density and long lifespan [1,2]. Corresponding energy storage mechanisms divide supercapacitor electrode materials into two types: carbon materials which store charge on the electrode/electrolyte interface via physical adsorption of ions (electrochemical double layer capacitance, EDLC), and transition metal oxides based on the fast reversible Faradaic redox reactions (also called pseudocapacitance) [3]. 

Recently, various carbon materials have been intensively studied for their high conductivity, good stability and relatively low cost, including activated carbon, porous carbon, hollow carbon, carbon nanotubes and graphene [4,5]. Among them, activated carbon (AC) with abundant micropores is promising for commercialization as an electrode material for supercapacitors due to its high surface area and low cost [6,7,8,9]. However, microporous AC with random pore size distribution usually suffers from limited ion-accessible surface area and low rate capability. Furthermore, to synthesize hierarchical porous carbon with controllable pore sizes, present techniques usually require diverse templates [10,11,12] and complex costly processes [13,14], some of which even cause unfavorable effects on the environment. To be precise, the ordered mesopores (2–50 nm) are believed to facilitate electrolyte diffusion [14,15]. The abundant volume of micropores (<2 nm) is considered to provide enough adsorbing sites [16], and macropores (<100 nm) have been shown to offer efficient nanoscale diffusion distance [17]. Bearing this in mind, it is desirable to develop new routines for obtaining activated carbon with hierarchical pore size distribution, high electrical conductance and hierarchical porosity.

Concerning the aforementioned challenge and additional factors such as cost of raw material and impact to the environment, renewable and engineering-level carbon electrodes derived from nature biochar waste have become a prospective choice. Inexpensive biomass, such as wood, agricultural residue and plants, could easily overcome the cost and environmental constraints mentioned above, thus, they have attracted much attention from researchers [18]. For instance, flexible fiber prepared from religiosa leaves demonstrated a capacitance of 3.4 F·g^−1^ in gel-polymer electrolyte [19], while sago bark through one-step pyrolysis showed a capacitance of 180 F·g^−1^ and good stability [20]. In spite of the ideal case, in which biomass carbon successfully combines the economy and sustainability of biowaste with superior electrochemical properties of nanomaterials, some of the activated biochar electrodes suffer in practice from limited rate capability and larger inner resistance, which can be attributed to random porous texture and disordered graphitic structure [21]. 

Herein, we report the synthesis of corncob-derived carbon with hierarchical porosity. Inheriting the special biogenetic textures of corncob and benefiting from proper activation process, the carbon materials produce abundant porous structures in various pore sizes from macro to micro scale. Through a low-cost and controllable carbonization process followed by KOH activation, the resulting pyrolyzed-activated carbon materials exhibit outstanding specific surface area (1471.4 m^2^·g^−1^), excellent electrical conductivity and high specific capacitance (293 F·g^−1^ at 1 A·g^−1^). Moreover, the fabrication of pyrolyzed-activated carbon-based supercapacitor enables the device to demonstrate remarkable energy and power densities (a maximum energy density of 20.15 Wh·kg^−1^ at a power densities of 500 W·kg^−1^) and excellent cycling stability (99.9% retention after 4000 cycles), showing great promise as a low-cost, approachable and high performance supercapacitor for energy storage application.

## 2. Materials and Methods 

### 2.1. Synthesis of Pyrolyzed Carbon Materials

Clean corncobs used in this work were collected from Aodong Agricultural Products Co., Ltd. (Hong Kong, China). Typically, the central pith (the soft part) was removed by mechanical treatment, remaining the hard outer woody ring blocks for the preparation of carbon materials. The obtained woody ring blocks were further cut into small pieces for pyrolysis process, during which these pieces were heated at 700 °C for 2 h in a tubular furnace under Ar protection. 

### 2.2. Preparation of Pyrolyzed-Activated Carbon Materials

For the convenience of the activation process, the as-obtained charcoal materials were further ground with a mortar to obtained powder. After that, 400 mg of KOH was firstly dissolved in 40 mL of ethanol and then 100 mg of charcoal was impregnated into the solution. The obtained suspension liquid was stirred at room temperature for 6 h and dried at 60 °C for 24 h. Next, the material was annealed at 700 °C under Ar atmosphere in a tubular furnace for 2 h. Finally, the collected materials were cleaned by 3 M HCl and deionized water for several times and dried at 60 °C for 24 h. The hierarchical porous pyrolyzed-activated carbon materials as mentioned were successfully obtained.

### 2.3. Preparation of Carbon Electrodes and Supercapacitor Device

80 mg of pyrolyzed-activated carbon materials, 10 mg of polyvinylidene fluoride (PVDF) and 10 mg of acetylene black were mixed and ground to prepare the supercapacitor electrodes. After ultrasound and stirring treatment, the as-obtained mixed slurry was coated on nickel foam, which was used as a current collector. After that, the as-prepared electrodes were dried in vacuum overnight at 120 °C. Finally, the carbon electrodes were obtained after a pressure treatment of 1.6 × 10^7^ Pa on the nickel foam. The mass loading of the pyrolyzed-activated carbon materials on the nickel foam was about 2–3 mg.

The supercapacitor device was assembled by applying two pyrolyzed-activated carbon electrodes with approximate mass. In addition, 6 M KOH solution was used as the electrolyte. TF4050 purchased from NKK Co., Ltd. (Tokyo, Japan) was used as the separator.

### 2.4. Characterization of Materials

Field emission scanning electron microscopy (FESEM, FEI Quanta 450, 20 kV, Beijing, China) and transmission electron microscopy (FETEM, JEOL JEM-2100, Shanghai, China, 200 kV equipped with an Oxford energy dispersive X-ray spectroscope (EDX), Shanghai, China) were applied to analyze the morphology of the corncob-derived carbon materials electrode materials. In addition, X-ray diffraction (Bruker D2 Phaser, 40 kV, 30 mA, Hong Kong, China) and X-ray photoelectron spectroscopy (XPS Physical Electronics PHI 5802, Hong Kong, China) were used to study the crystalline and composition information of the material. Thermogravimetric analysis was carried out to analyze the pyrolysis process (TGA, TA Instruments, SDT Q600, Shanghai, China) from room temperature to 750 °C with a temperature rise of 5 °C/min and a nitrogen flow of 20 sccm. N_2_ adsorption-desorption measurements on a surface area analyzer (Quantachrome Nova 1200e, Hong Kong, China) at 77 K was used to study the porous structures of the sample, with Brunauer-Emmett-Teller (BET) method calculating the surface area and Density Functional Theory (DFT) analyzing the pore-size distribution. 

### 2.5. Electrochemical Measurements

The electrochemical properties of the as-obtained carbon materials were tested using them as a single electrode in a three-electrode system. To be specific, a Hg/HgO electrode was the reference electrode, platinum foil was the counter electrode and 6 M KOH solution was the electrolyte. Cyclic voltammetry (CV), galvanostatic charge/discharge (GCD) and electrochemical impedance spectroscopy (EIS) tests were conducted via an electrochemical workstation CHI-660e. In addition, the supercapacitor device was tested under cycling measurements by GCD mode (BTS 6.0, Neware Co., Ltd., Shenzhen, China). 

For GCD tests, the specific capacitances were calculated as:

(1) $$C_{s}=\frac{it}{m\Delta V}$$

where *C*_s_ is the specific capacitance (F·g^−2^), *i* is the current (A), *t* is the discharge time (s), *m* is the mass of the electrode material (g) and Δ*V* is the potential window (V).

For CV tests, the specific capacitances are calculated as:

(2) $$C_{s}=\int^{\text{​}}\frac{I\left(V\right)dV}{v\Delta V}$$

where *C*_s_ is the specific capacitance (F·g^−1^), *I(V)* is the current density response (mA·g^−1^), *v* is the scan rate (V·s^−1^) and Δ*V* is the potential window (V).

Energy and power densities are calculated based on the following equations:

(3) $$E=\frac{C_{s}}{2\Delta V^{2}}$$

(4) $$P=\frac{E}{t}$$

where *E* is the energy density (Wh·kg^−1^) and *P* is the power density (W·kg^−1^).

## 3. Results and Discussion

### 3.1. Structure Analysis

**Figure 1:**
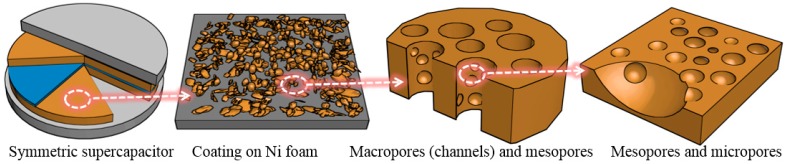
Schematic illustration for the hierarchical porous structures of corncob-derived carbon.

Figure 1 illustrates the hierarchical porous structures of corncob-derived carbon materials by step-by-step amplification. The idea when preparing the hierarchical porous structure of pyrolyzed-activated carbon materials is to combine the numerous channels and pores inherent to the nature of corncob with the additional pores prepared from the carbonization and activation process. By the facile and cost-effective two-step processes, pore sizes of the material are expected to cover macro (10 μm to 100 nm), meso (50 to 10 nm) and micro (<2 nm) scales, which will be verified and analyzed systemically in a later part. The woody ring utilized in the research is mainly composed of cellulose and some other components, for example, hemicellulose and lignin [21]. After the pyrolysis in tubular furnace, carbon materials are obtained with macro and meso pores inherited from natural corncob. Then, the following chemical activation treatment further generates microporous structure and high specific surface area, finally generating a hierarchical porosity.

**Figure 2:**
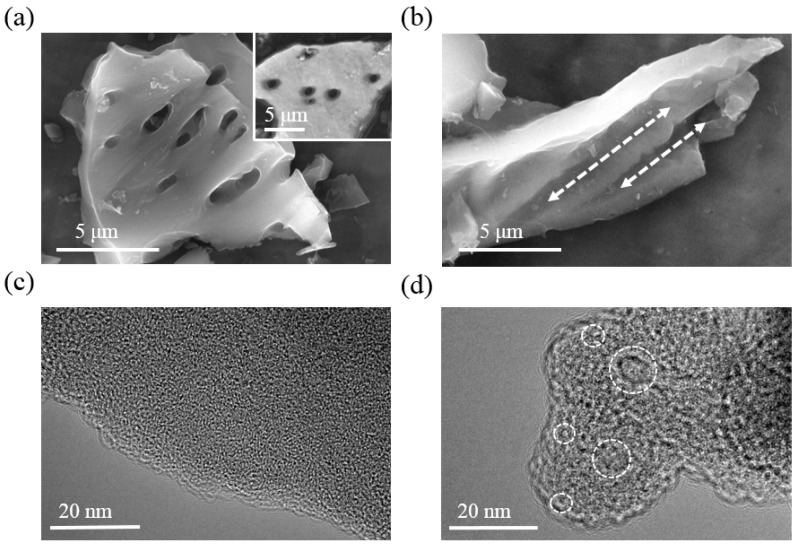
SEM images of pyrolyzed-activated carbon (**a**) macropores and (**b**) channels inherited from natural corncob materials, TEM images of corncob-derived carbon materials (**c**) before and (**d**) after activation, inset of (**a**) is the SEM image of natural corncob.

The hierarchical porosity of pyrolyzed-activated carbon materials is systemically verified by FESEM and TEM images. SEM image of the as-obtained pyrolyzed-activated carbon materials (Figure 2a) shows ordered macropores on the surface of obtained carbon materials. Compared with the inset, which represents the SEM image of natural corncob, it is found that these uniform macropore structures are maintained during the pyrolysis and activation steps. As the particle size of as-obtained carbon materials is in the range of tens of micrometers, the SEM image in Figure 2b exhibits some macro-size channels in the same order of magnitude, with a diameter of around 1 μm (which is in agreement with the size in Figure 2a and 10 μm long, which is also very likely to be inherited from the natural textures of corncob [21]. Furthermore, TEM images before and after the activation process are compared in Figure 2c,d. It is obvious that the pyrolyzed-activated carbon materials in Figure 2d exhibit numerous mesopores and micropores (marked in white circles), fewer of which are observed in Figure 2c. These abundant meso- and micropores contribute to a high surface area and prior capacitance behaviors [22,23]. 

**Figure 3:**
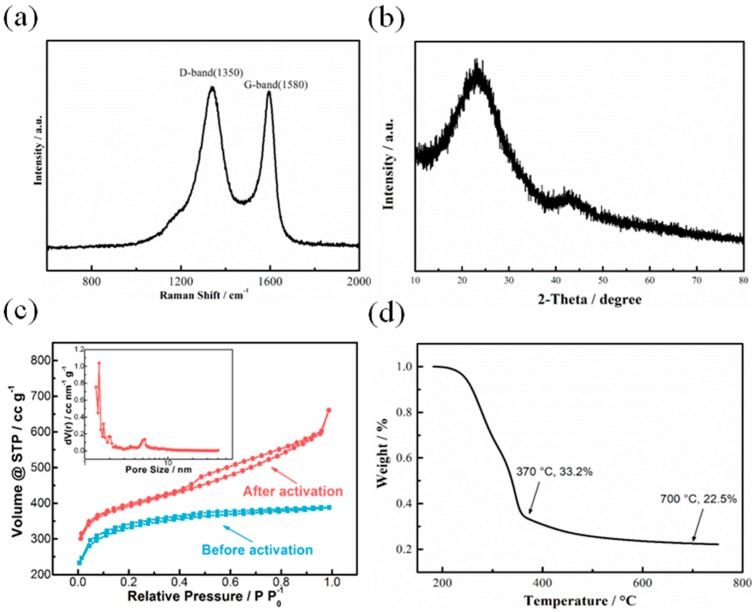
(**a**) Raman spectrum and (**b**) XRD pattern of pyrolyzed-activated carbon materials; (**c**) N_2_ gas adsorption-desorption curve of pyrolyzed carbon materials before and after activation; (**d**) TGA curve of corncob pyrolysis, the inset of (**c**) is the corresponding pore size distribution curve of pyrolyzed-activated carbon materials.

Raman, XRD, BET and TG tests are applied to study the material properties of corncob-derived carbon materials. Figure 3a presents the Raman spectrum, with two separated peaks at 1350 and 1580 cm^−1^, which correspond to the D band and G band, respectively. The peak intensity of these two bands, as an index to suggest the crystallinity of carbon materials, is calculated to be 0.97. In addition, the XRD pattern of pyrolyzed-activated carbon materials in Figure 3b exhibits two broad peaks at 25° and 43°, suggesting the generation of carbon materials. The N_2_ gas adsorption-desorption isotherm experiment is carried out to study the porosity of the corncob-derived carbon materials. In Figure 3c, a hysteresis loop is clearly observed in the cures of activated materials, indicating that the material is mainly composed of micro- and mesopores. By comparison, the adsorption-desorption curve before activation process indicates that the materials consist of macropores, which proves the generation of micropores and mesopores in the carbon materials during the KOH activation process. Furthermore, the inset of Figure 3c further demonstrates the hierarchical porous structure of corncob-derived carbon materials, with a majority of micropores and abundant macrospores, which is in accordance with the aforementioned HRTEM images. With the BET model, it is worth noting that the specific surface area is calculated to be 1471.4 m^2^·g^−1^, suggesting excellent porosity and promising application as capacitive materials. Lastly, the TG curve in Figure 3d demonstrates the carbonization process of corncob-derived carbon, with 22.5% of mass remaining at the final temperature.

### 3.2. Electrochemical Behaviors

**Figure 4:**
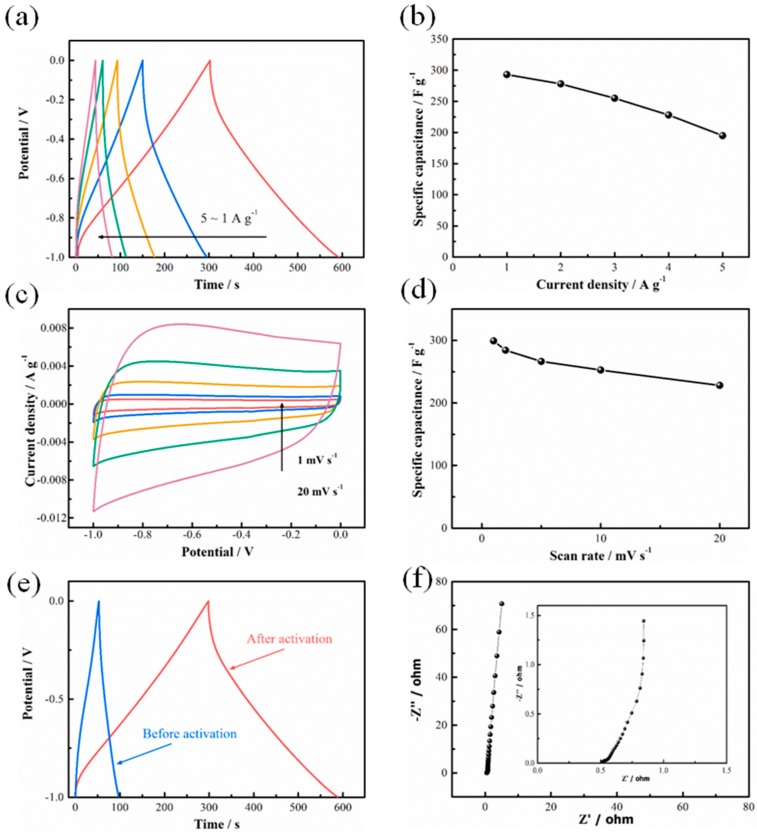
Electrode materials electrochemical tests of corncob-derived carbon materials (**a**) GCD curves of pyrolyzed-activated carbon materials in current densities of 1, 2, 3, 4 and 5 A·g^−1^ and (**b**) their rate capabilities; (**c**) CV curves of pyrolyzed-activated carbon materials in different scan rates at 1, 5, 10, 15 and 20 mV·s^−1^; (**d**) corresponding rate capabilities; (**e**) GCD curves of corncob-derived carbon materials before and after activation process; (**f**) Nyquist plots of pyrolyzed-activated carbon materials with an enlarged high frequency region.

Due to the high specific area and well-designed hierarchical porous structures, the as-obtained pyrolyzed-activated carbon materials are applied as EDLC electrodes for supercapacitors. The electrode electrochemical performances of pyrolyzed-activated carbon materials are tested by CV, GCD and EIS by a three-electrode system, with 6 M KOH solution as the electrolyte. Figure 4a plots the GCD curves of pyrolyzed-activated carbon materials in various current densities. It is noted that all the curves maintain ideal linear shapes. Also, charge and discharge parts keep a symmetric relation with tiny IR drop, indicating excellent stability and reversibility of the material. As plotted in Figure 4b, at the current densities of 1, 2, 3, 4 and 5 A·g^−1^, 293, 278, 255, 228 and 195 F·g^−1^ are obtained, respectively. Furthermore, CV curves of pyrolyzed-activated carbon materials are plotted as Figure 4c, which exhibit quasi-rectangular shapes, demonstrating ideal supercapacitive behaviors. Using Equation (2), the specific capacitances are calculated to be 299, 284, 266, 252 and 227 F·g^−1^ at scan rates of 1, 2, 5, 10 and 20 mV·s^−1^, as shown in Figure 4d. Furthermore, the GCD curves of carbon materials before and after KOH activation are compared in Figure 4e. The obvious enlarged discharge time of pyrolyzed-activated carbon materials proves the advantages of the hierarchical porous structure, which leads to increased specific surface area (thus higher EDLC capacitance), easier electrolyte infiltration, faster ion transportation and higher conductivity. Lastly, Nyquist cure of pyrolyzed-activated carbon materials in Figure 4f demonstrates a very small arc in the high-frequency region and a perfect straight line in the low-frequency region, proving the typical capacitive response and excellence electrochemical performance of the obtained materials.

**Figure 5:**
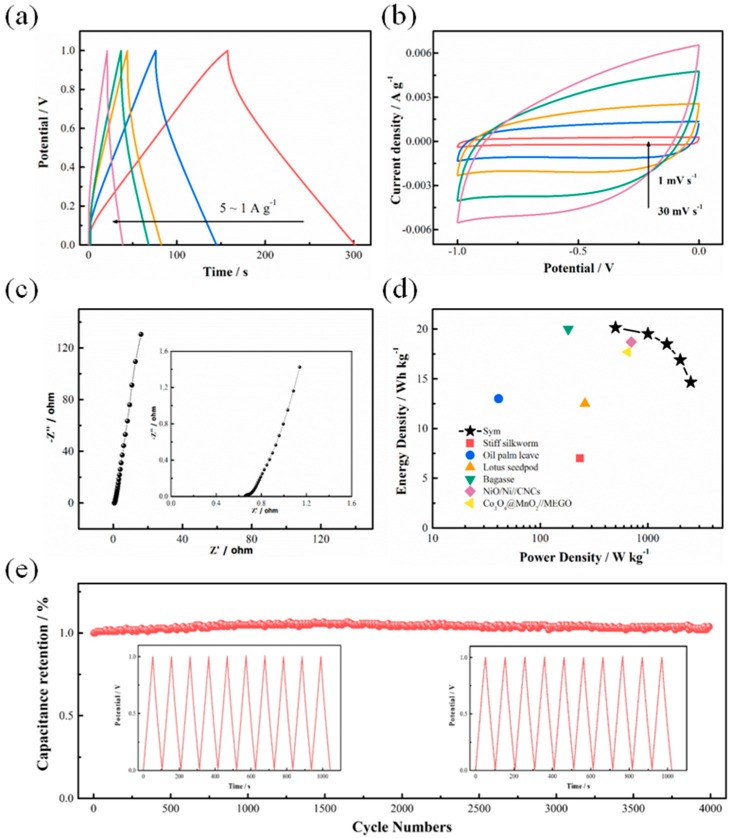
Electrochemical performances of corncob-derived carbon symmetric device: (**a**) GCD curves at current densities of 1, 2, 3, 4 and 5 A·g^−1^; (**b**) CV curves at different scan rates of 1, 5, 10, 20 and 30 mV·s^−1^; (**c**) Ragone plot (the inset shows the enlarged high frequency region); (**d**) Nyquist plots (energy density vs. power density); and (**e**) cycling test of corncob-derived carbon symmetric device in 2 A·g^−1^; 6 M KOH, inset is the GCD curves for the first and last ten cycles.

To understand the practical application of corncob-derived carbon materials, two electrodes with comparable mass loading are selected to assemble a symmetric supercapacitor. As noted in Figure 5a,b, the as-obtained device exhibits good EDLC behaviors. It is worth noting that all GCD curves remain symmetric, and CV curves retain similar shapes, demonstrating good capacitive properties. In addition, the EIS test in Figure 5c exhibits the low charge transfer resistance (~0.02 Ω) and solution resistance (~0.45 Ω) of the device. For a practical application, the Ragone plot in Figure 5d shows the highest energy density, at 20.15 Wh·kg^−1^, at a power density of 500 W·kg^−1^, demonstrating superior performance compared with previous reports such as stiff silkworm (234 W·kg^−1^ at 7.9 Wh·kg^−1^) [24], oil palm leaf (41 W·kg^−1^ at 13 Wh·kg^−1^) [25], lotus seedpod (260 W·kg^−1^ at 12.5 Wh·kg^−1^) [26], and bagasse (182 W·kg^−1^ at 20 Wh·kg^−1^) [27]; even some asymmetric devices Co_3_O_4_@MnO_2_//MEGO (650 W·kg^−1^ at 17.7 Wh·kg^−1^) [28] and Co_3_O_4_@MnO_2_//MEGO (400 W·kg^−1^ at 21.1 Wh·kg^−1^) [29]. What’s more important, in Figure 5e, the specific capacitance of the device shows almost no decay after 4000 cycles, demonstrating excellent stability of corncob-derived carbon materials.

## 4. Conclusions

In summary, corncob-derived carbon materials were synthesized through a simple and cost-effective process from corncob for supercapacitor application. Inheriting from nature the porous texture of the biomass precursor, the pyrolysis and activation processes generate hierarchical porous structures, with a scale ranging from macro, to meso and micro levels. SEM, TEM and pore size distribution results systemically prove the porosity of corncob-derived carbon materials, with a high BET surface area of 1471.4 m^2^·g^−1^. For electrochemical evaluation, the specific capacitance of pyrolyzed-activated carbon materials electrode is calculated to 299 F·g^−1^ at 1 A·g^−1^. While for the supercapacitor device, an energy density of 20.15 Wh·kg^−1^ is obtained at a power density of 500 W·kg^−1^, and 99.9% of the capacitance retention is obtained after 4000 cycles. These results suggest promising potential for supercapacitor application. The concept of this work, carbonization based on biomass natural textures, is expected to be further employed to prepare other environmentally friendly energy storage materials.
